# Case Report: First case of a pediatric laryngeal inflammatory rhabdomyoblastic tumor—diagnostic dilemma and insights into the conservative management of a residual lesion

**DOI:** 10.3389/fped.2025.1489754

**Published:** 2025-06-11

**Authors:** M. S. Nadhirah, A. Hamidah, A. A. Aishah Harizah, R. M. Z. Reena, T. Y. Kew, M. B. Marina

**Affiliations:** ^1^ Department of Otorhinolaryngology—Head and Neck Surgery, Faculty of Medicine, Universiti Kebangsaan Malaysia, Kuala Lumpur, Malaysia; ^2^ Department of Otorhinolaryngology—Head and Neck Surgery, Hospital Canselor Tuanku Muhriz, Bandar Tun Razak, Cheras, Kuala Lumpur, Malaysia; ^3^ Department of Paediatrics, Faculty of Medicine, Universiti Kebangsaan Malaysia, Kuala Lumpur, Malaysia; ^4^ Department of Pathology, Faculty of Medicine, Universiti Kebangsaan Malaysia, Kuala Lumpur, Malaysia; ^5^ Department of Radiology, Faculty of Medicine, Universiti Kebangsaan Malaysia, Kuala Lumpur, Malaysia

**Keywords:** histiocyte-rich rhabdomyoblastic tumor, inflammatory leiomyosarcoma, inflammatory rhabdomyoblastic tumor, rhabdomyosarcoma, larynx

## Abstract

An inflammatory rhabdomyoblastic tumor (IRMT), formerly known as a histiocyte-rich rhabdomyoblastic tumor (HR-RMT), has been identified as a skeletal muscle neoplasm exhibiting a mild clinical course. However, recent studies suggested that an HR-RMT has significant pathological and genetic overlap with inflammatory leiomyosarcoma, leading to the proposal of the newly defined entity, IRMT. We report the first case of an IRMT affecting the larynx in an adolescent girl who presented with a 3-month history of hoarseness. Endoscopic examination revealed a left paraglottic mass, which was surgically removed. No disease progression was observed over a 4-year follow-up. We discuss the diagnostic and management challenges, especially the role of adjuvant therapies in pediatric patients. This case highlights the importance of a precise diagnosis and careful management and emphasizes the need to increase awareness of IRMT.

## Introduction

Mesenchymal tumors are regarded as one of the most challenging fields of diagnostic pathology owing to their scarcity and intrinsic complexity, with frequent violations of malignancy diagnostic criteria (1). Another factor leading to diagnostic inaccuracy is the intricacy of the diagnostic process that demands integration of microscopic, immunophenotypical, and molecular features. The WHO Classification of Soft Tissue Tumors and Bone contributes to refining the classifications and standardizing the diagnosis to improve the therapeutic outcomes (1). Some mesenchymal tumors are categorized as “intermediate” either due to their locally aggressive behavior or low tendency for metastasis (1). However, tumors with skeletal muscle differentiation are still classified as benign rhabdomyomas and malignant rhabdomyosarcoma (RMS) (1). Martinez et al. were the first to report a histologically distinctive histiocyte-rich rhabdomyoblastic tumor (HR-RMT). It has skeletal muscle differentiation but does not fit into the benign or malignant classification (2). An HR-RMT is characterized by slow-growing, well-circumscribed nodular masses in review surrounded by fibrous tissue containing lymphoid aggregates with striking proliferation of non-neoplastic histiocytes obliterating the underlying rhabdomyoblastic tumor. Despite having “malignant” morphological characteristics such as eosinophilic cytoplasm, large nuclei, and prominent nucleoli, an HR-RMT exhibits low mitotic activity and a lack of morphological atypia (2). It has focal positivity for at least one skeletal muscle marker (myoD1 or myogenin), with diffuse desmin positivity (2). HR-RMTs have been reported in the neck, thoracic wall, abdominal wall, and upper and lower extremities with favorable clinical outcomes and a low risk of distant metastasis (2, 3). Inflammatory leiomyosarcoma (ILMS) was first classified as a malignant tumor of smooth muscle in the latest WHO Classification of Soft Tissue Tumors and Bone 2020, however, ILMSs behave less aggressively than leiomyosarcomas (1). Cloutier et al. studied the clinicopathological, immunohistochemical, and genetic features of cases that were previously classified as ILMS and HR-RMT (4). Interestingly, both tumors have overlapping morphological and immunohistochemical features, particularly the presence of immunohistochemical evidence of skeletal muscle marker expression in ILMS and chromosomal microarray studies showed concurrence near-haploidization events (4). The study concluded ILMS and HR-RMT are the same entity and proposed a reclassification of the nomenclature to “inflammatory rhabdomyoblastic tumor” (IRMT) to precisely describe the notable morphological and immunochemical features of both tumors.

We report a case of a laryngeal IRMT in an adolescent girl presenting with hoarseness. We discuss the differential diagnosis and therapeutic challenge in managing this rare tumor in the larynx.

## Case report

**Figure 1:**
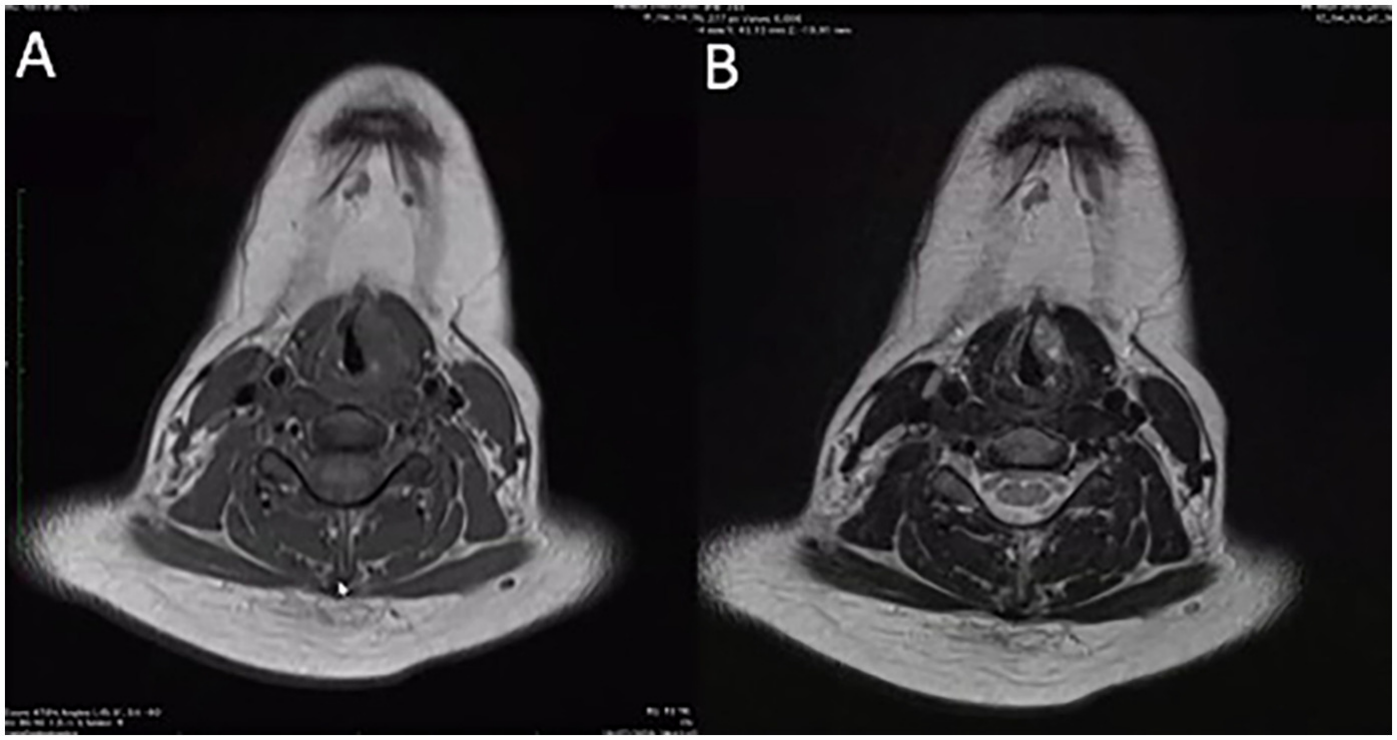
Contrast magnetic resonance imaging of the head and neck. **(A)** A heterogenous lesion located at the anterior 2/3rd of left paraglottic space measuring 1.9 cm × 0.8 cm × 1.5 cm, that was low signal on T1, **(B)** heterogenous hyperintense on T2, and enhanced on post-contrast. The lesion abuts the left thyroid lamina without any radiological evidence of erosion.

A 13-year-old girl presented to the outpatient Department of Otorhinolaryngology with a complaint of persistent hoarseness for 3 months. There was no history of voice misuse, upper respiratory tract infection, night sweats, or weight loss. There was no history of difficulty in breathing, dysphagia, or aspiration symptoms. She has no family history of head and neck malignancy. Clinical examination revealed a grade 2 dysphonia, with the main component on the Grade, Roughness, Breathiness, Asthenia, Strain (GRBAS) scale being strain. There was no audible stridor, and she was not in respiratory distress. The laryngeal framework was normal, and there was no cervical lymphadenopathy. A flexible nasopharyngolaryngoscopy examination showed a submucosal mass over the left paraglottic space with smooth overlying mucosa, medializing the left true vocal fold and obliterating the left ventricle. There was limited mobility of the left vocal fold. Her nasal and ear examinations were normal. Other system examinations were unremarkable. Contrast magnetic resonance imaging (MRI) of the neck was performed and showed a heterogenous lesion at the left anterior 2/3rd of the true vocal fold measuring 1.9 cm × 0.8 cm × 1.5 cm, which had low signal on T1, was heterogenous hyperintense on T2, and was enhanced on post-contrast (Figure 1). The mass was abutting the left thyroid cartilage without causing any erosion. There were multiple non-enhancing subcentimeter cervical nodes with preserved fatty hila seen at levels I, II, and III, with the largest node on the left at level II measuring 0.9 cm in diameter.

**Figure 2:**
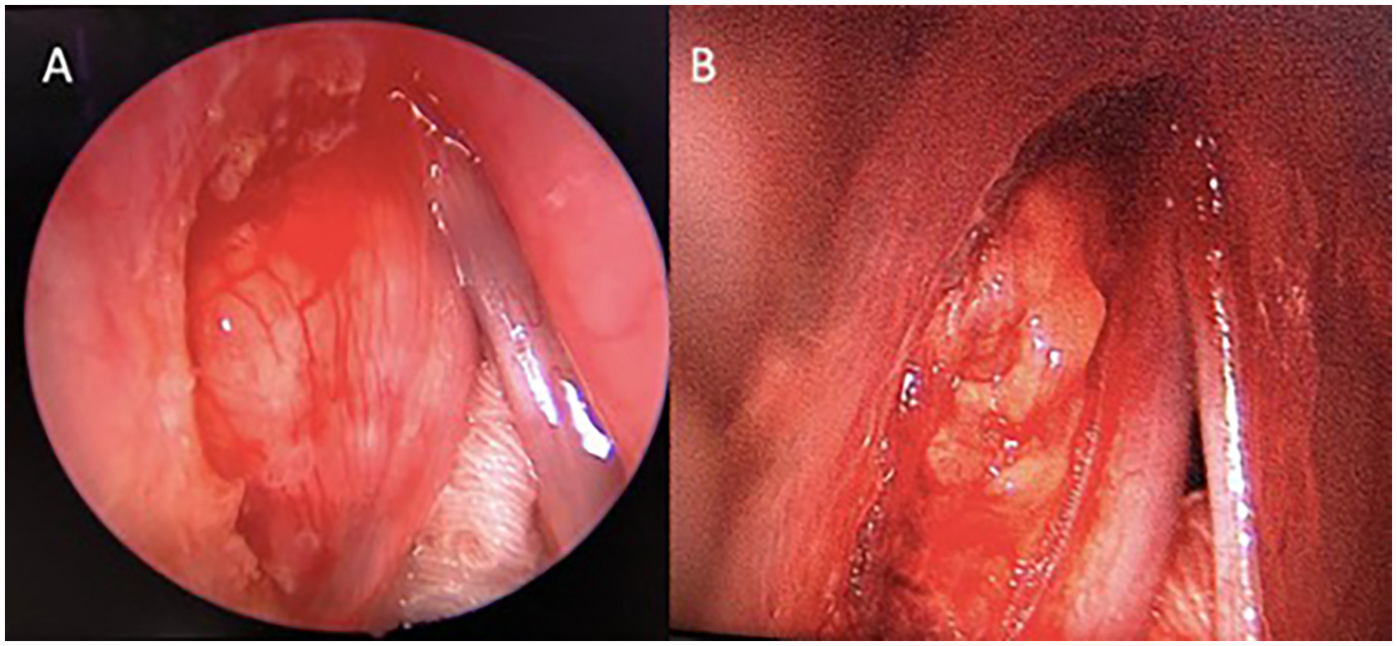
Direct laryngoscopy view of the larynx. **(A)** The left false cord was excised to gain a better view of the mass. **(B)** A lobulated mass occupies the left paraglottic space, extending medially to the thyroarytenoid muscle and anterolaterally to the thyroid lamina, with no erosion.

**Figure 3:**
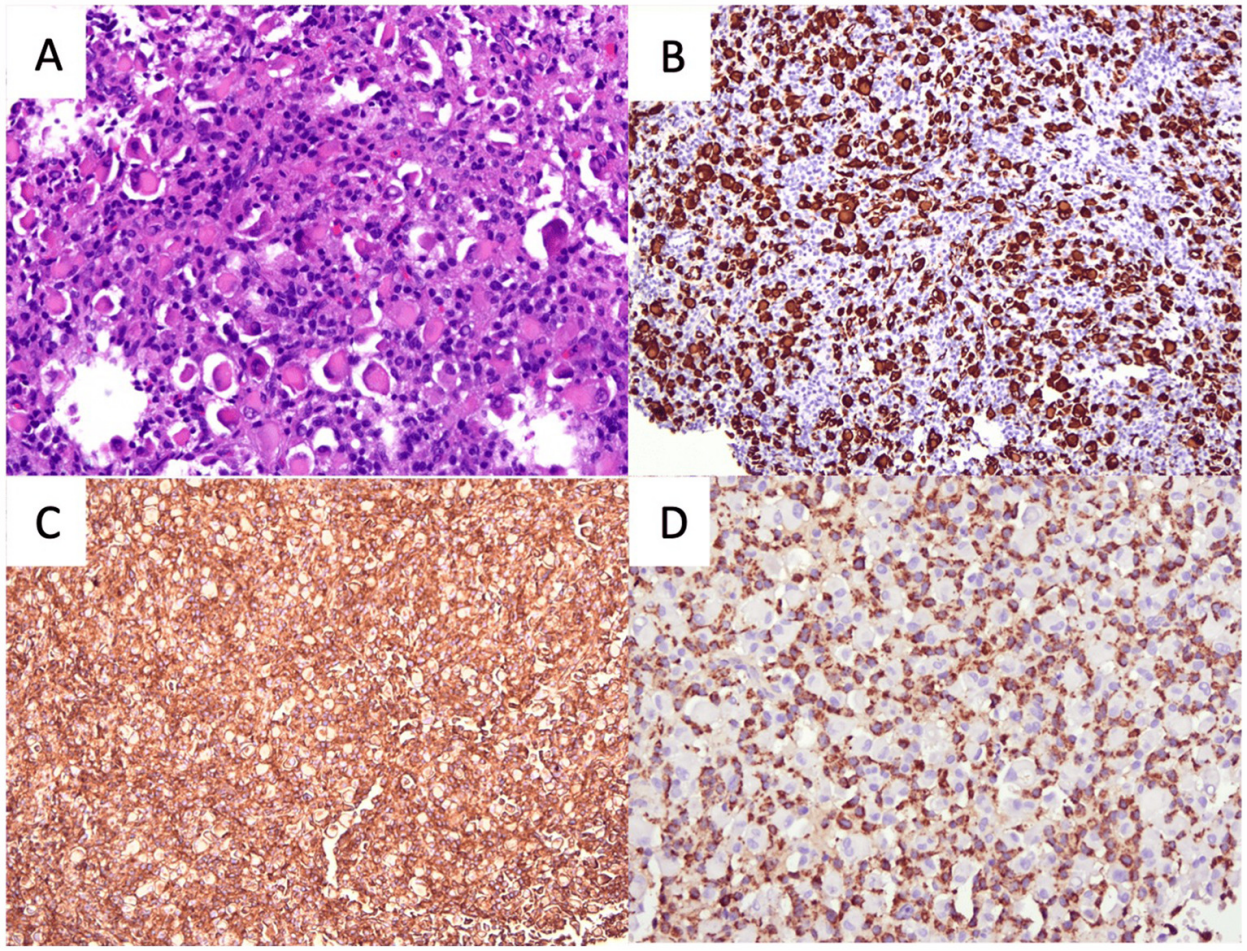
The histopathological features of the lesion. **(A)** Image of H&E staining showing large polygonal rhabdoid-like cells, interspersed with fairly monomorphous small to intermediate rounded cells. **(B)** Desmin immunohistochemistry staining showing the rhabdoid-like cells. **(C)** CD99 immunohistochemistry staining showing strong diffuse positivity and **(D)** CD68 immunohistochemistry staining showing small to intermediate rounded cells (all figures magnified ×20).

Subsequently, she underwent a direct laryngoscopy, examination under anesthesia, and endoscopic laryngeal microsurgery. An intraoperative examination showed a smooth submucosal mass with normal overlying mucosa over the left paraglottic space, displacing the left true vocal fold medially. Initially, the medial part of the false vocal fold was excised for better visualization. Then, the overlying mucosa of the left paraglottic space was incised using a CO_2_ laser, exposing the capsule of the mass (Figure 2). Unfortunately, the capsule was unintentionally ruptured. The mass was carefully removed from the surrounding structure in the paraglottic space. The left recurrent laryngeal nerve (RLN) was visualized beneath the mass and then preserved. The patient recovered well post-operatively and was discharged the next day. The histopathological examination of the mass revealed tumor tissue comprising large polygonal rhabdoid-like cells, interspersed with fairly monomorphous small to intermediate rounded cells. The overall features were interpreted as an intermediate to high-grade lesion, suggestive of a sarcoma with histiocytic differentiation and favoring rhabdomyosarcoma (Figure 3A). An ancillary study with immunohistochemistry showed strong and diffuse reactivity for desmin and CD 99 (Figures 3B,C). Many of the tumor cells also expressed histiocytic markers such as CD 68 (Figure 3D) and CD 31 (not shown). Upon consultation, stained slides and a tissue block were sent to the Mayo Clinic Cancer Center for a chromosomal microarray test, however, the examination was not performed due to insufficient DNA.

A multidisciplinary discussion among the resident pediatric oncologist, otorhinolaryngology surgeon, and pathologist concluded that the tumor did not show typical features of either benign rhabdomyoma or malignant RMSs. A revised diagnosis of an intermediate to high-grade lesion with uncertain malignant potential was made. A second opinion was sought from international pathology experts who suggested a diagnosis of HR-RMT. MRI of the whole body showed no other abnormal signal intensity in the rest of the body to suggest any metastatic disease. Thus, no systemic therapy of chemotherapy or radiotherapy was administered for her condition after further evaluation by the pediatric oncology team.

**Figure 4:**
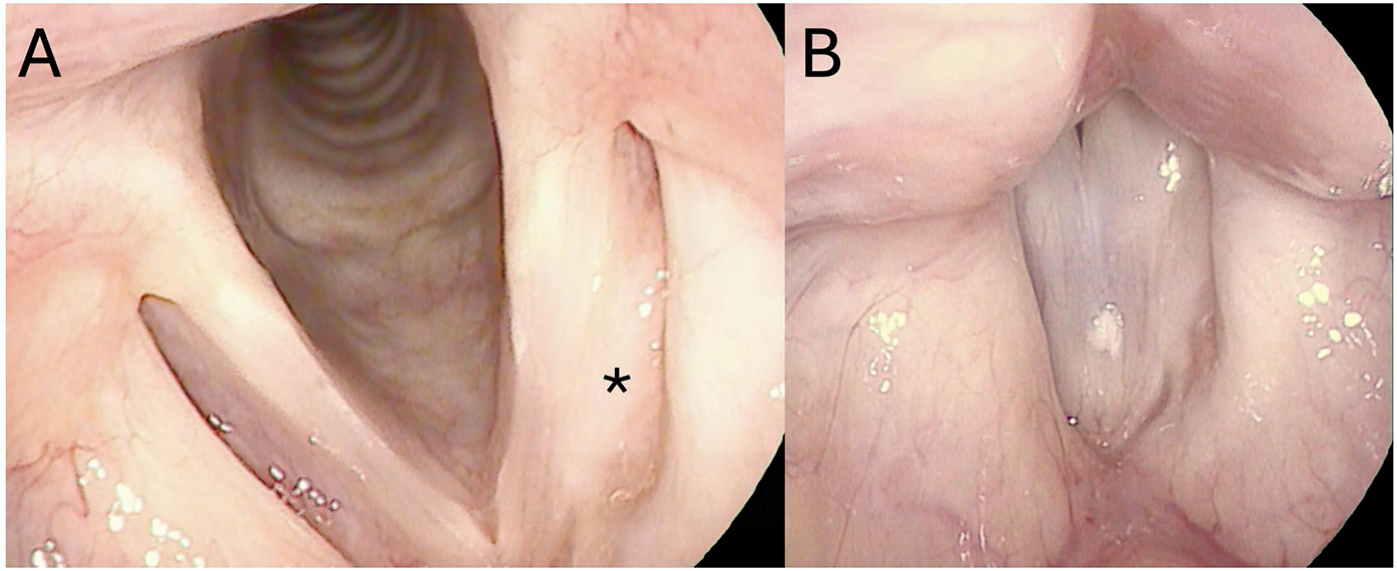
Post-operative surveillance laryngostroboscopic examination showing fullness over the left paraglottic space (black asterisk), which did not obliterate the ventricle or laryngeal inlet **(A)**. Upon phonation **(B)**, there was complete glottal closure with normal mucosal waves bilaterally.

Repeat MRI of the neck 6 months post-operatively showed persistence of the left paraglottic lesion, which was smaller in size (measuring 0.7 cm × 0.3 cm). In view of the patient being asymptomatic and no evidence of local recurrence on endoscopy examinations (Figure 4), we decided to monitor the disease clinically and perform surveillance MRI every 6 months. Up to 4 years of follow-up, the 6-monthly surveillance MRI showed that the size of the left paraglottic lesion remained unchanged and there was no radiological evidence of disease progression, local spread, or distant metastasis. The patient remained asymptomatic, with significant improvement of both subjective and objective voice parameters.

## Discussion

IRMT is a recently described neoplasm with skeletal muscle differentiation that has intermediate morphological and immunochemical features and uncertain malignant potential (4). Despite not yet being recognized in the latest 2020 WHO Classification of Soft Tissue Tumors and Bone (5th edition), awareness of IRMTs is increasing among pathologists owing to their unique characteristics. The newly proposed classification indicates striking morphological and immunohistochemical similarities between previously described HR-RMTs and ILMS. Known for its distinct features of non-neoplastic histiocytes proliferation obliterating the underlying rhabdomyoblastic tumor, diffuse desmin positivity, and focal positivity to at least one skeletal muscle marker among SMA, MYOD1, and myogenin, an IRMT presents with indolent clinical behavior with unknown risk of metastasis. IRMTs are frequently reported as slow-growing soft tissue masses arising from the skeletal muscles in the lower extremities and trunk, which exhibit indolent behavior. They commonly affect young to middle-aged male patients, with the youngest case of IRMT reported in a 5-year-old boy who presented with an intramuscular mass in the arm (5).

The present case demonstrates the characteristic microscopic and immunohistochemical features of an HR-RMT, as previously described by Martinez et al. (2), and the latest proposed classification of an IRMT by Cloutier et al. (4). The differential diagnoses for IRMT include spindle cell/sclerosing RMS and angiomatoid fibrous histiocytoma (AFH). AFHs share certain characteristic morphology with IRMTs, but desmin positivity is only found in 50% of cases (3). AFHs have characteristic EWSR1-CREB1 gene fusions, which are not reported in HR-RMTs and IRMTs. Spindle cell RMSs lack intratumoral histiocytic infiltration and demonstrate aggressive behavior in adult patients (3). Sclerosing RMSs exhibit primitive round cells with a high nuclear grade in sclerotic hyalinized collagenous stroma (3). Furthermore, both spindle cell RMSs and sclerosis RMSs harbor the MYOD1 L122R mutation, which has not been observed in any IRMT cases (3).

To the best of our knowledge, this is the first reported case of an IRMT in the larynx. The presenting symptoms of a laryngeal tumor are nonspecific, depending on the tumor’s size, location, and pace of growth. Patients may complain of dysphonia, globus sensation, reduced effort tolerance, and upper airway obstruction (6). Small tumors may be asymptomatic, and thus it is difficult to diagnose them in the early stages. Our patient only presented with hoarseness. This non-specific symptom has broad differential diagnoses, ranging from the more common laryngitis and benign vocal fold lesions to a malignant laryngeal lesion. A laryngoscopy examination is paramount to provide a dynamic view of the larynx and locate the mass. Imaging, such as MRI, although non-specific, helps in showing the extension of the mass and its relation to the surrounding structures, which are crucial in pre-operative planning. However, submucosal laryngeal tumors in particular remain a clinical and radiological challenge. A laryngoscopy examination and imaging characteristics are insufficient tools, and a biopsy is mandatory for diagnosis (6). A karyotyping analysis would show hypodiploidy with retained 5, 21, and 22 in an IRMT. A genetic study is important to identify the near-haploid pattern with loss of heterozygosity, a genetic hallmark of an IRMT.

The majority of the reported IRMT cases have indolent clinical behavior and a favorable prognosis (7). Albeit rare, progression of IRMTs to high-grade RMSs has been reported (4, 8, 9), indicating the potential for more aggressive behavior in exceptional instances. Diminished histiocytes with an overgrowth of monomorphic rhabdomyoblasts, monomorphic spindle cell morphology with pleomorphic rhabdomyoblasts, and undifferentiated spindle cell and epithelioid sarcoma are the features observed (9). Dehner et al. demonstrated widespread loss of heterozygosity with retained heterozygosity of chromosomes 5 and 20, and most cells displayed alterations in tumor suppressor genes, especially CDKN2A and CDKN2B (9). These findings support that a patient with an IRMT may have a variable clinical course, with underlying additional genetic mutations affecting its metastasis potential and treatment response (9). In our case, there was no histological evidence of a high-grade transformation suggestive of rhabdomyosarcoma, which has been associated with more aggressive clinical behavior in previously reported cases (9). In addition, the confined anatomical compartment of the larynx, which is surrounded by cartilage and dense connective tissue, may have limited the potential for local invasion or aggressive spread. The tumor was well-circumscribed and encapsulated, further supporting a localized growth pattern rather than infiltrative behavior. The patient's young age and presumed immunocompetent state may also have influenced the indolent course, as younger individuals with IRMTs have been reported to experience more favorable outcomes, potentially due to more effective immune surveillance.

Currently, there is no standard treatment for IRMTs. Most of the cases were treated with wide local excision alone (7). Excision of a submucosal tumor in the larynx particularly poses a great challenge. Excising the tumor while preserving the surrounding structures is paramount to avoid devastating complications such as glottic stenosis and RLN injury. The role of adjuvant treatment is still arguable and reserved for cases with positive surgical margins or RMS transformation (3, 8).

Considering the rarity of IRMTs and the majority of the cases reporting indolent clinical behavior, the role of adjuvant radiotherapy or chemotherapy for our patient is highly debatable. Adjuvant treatments carry risks of developing treatment-related complications, including second primary malignancy and infertility, which pose a huge dilemma when treating an adolescent girl. Thus, after a careful multidisciplinary discussion including the parents, we decided to perform a surveillance laryngoscopy examination and MRI every 6 months. To date, 4 years post-surgery, the patient remains asymptomatic and there is no evidence of tumor recurrence and distant metastasis on serial endoscopic examination and imaging.

In conclusion, a laryngeal IRMT presents significant diagnostic and management challenges due to its rarity and its overlapping histological features with other neoplasms, such as rhabdomyosarcoma and ILMS. The decision to forgo adjuvant therapy was based on the tumor's indolent behavior and low risk of metastasis, with ongoing monitoring showing no recurrence or progression over 4 years. This case highlights the importance of a precise diagnosis and careful management and emphasizes the need to include IRMTs in the next WHO Classification of Soft Tissue Tumors and Bone. A more precise classification for this rare neoplasm will aid in a better diagnosis and understanding of it. Incorporating this newly defined pathological entity would enhance diagnostic accuracy and guide treatment strategies. With their unique characteristics and the increasing awareness of IRMTs, further studies on their genetic pathogenesis are also crucial to understand the biological behavior of this tumor and for any potential treatment recommendations.

## Data Availability

The original contributions presented in the study are included in the article/Supplementary Material, further inquiries can be directed to the corresponding author.
